# A novel germline gain-of-function HIF2A mutation in hepatocellular carcinoma with polycythemia

**DOI:** 10.18632/aging.102967

**Published:** 2020-04-01

**Authors:** Jiong Yu, Xiaowei Shi, Chunzhang Yang, Petra Bullova, Christopher S. Hong, Cody L. Nesvick, Pauline Dmitriev, Karel Pacak, Zhengping Zhuang, Hongcui Cao, Lanjuan Li

**Affiliations:** 1 State Key Laboratory for the Diagnosis and Treatment of Infectious Diseases, National Clinical Research Center for Infectious Diseases, Collaborative Innovation Center for Diagnosis and Treatment of Infectious Diseases, The First Affiliated Hospital, College of Medicine, Zhejiang University, Hangzhou City, China; 2 Surgical Neurology Branch, National Institute of Neurological Disorders and Stroke (NINDS), National Institute of Health (NIH), Bethesda, MD 20892, USA; 3 Department of Molecular Medicine, Institute of Virology, Slovak Academy of Sciences, Bratislava, Slovakia

**Keywords:** hypoxia-inducible factors, mutation, germline, Von Hippel-Lindau, hepatocellular carcinoma

## Abstract

Hypoxia-inducible factors (HIFs) regulate oxygen sensing and expression of genes involved in angiogenesis and erythropoiesis. Polycythemia has been observed in patients with hepatocellular carcinoma (HCC), but the underlying molecular basis remains unknown. Liver tissues from 302 HCC patients, including 104 with polycythemia, were sequenced for *HIF2A* mutations. A germline *HIF2A* mutation was detected in one HCC patient with concurrent polycythemia. Three additional family members carried this mutation, but none exhibited polycythemia or were diagnosed with HCC. The gain-of-function mutation resulted in a HIF-2α protein that was transcribed normally but resistant to degradation. HIF-2α target genes *EDN1*, *EPO*, *GNA14*, and *VEGF* were significantly upregulated in the tumor bed but not in the surrounding liver tissue. Polycythemia resolved upon total resection of the tumor tissue. This newly described *HIF2A* mutation may promote HCC oncogenesis.

## INTRODUCTION

Appropriate cellular responses to oxygen levels are essential for tissue homeostasis. Hypoxia-inducible factors (HIFs) are evolutionarily-conserved proteins that govern cellular response to low oxygen tension by initiating the transcription of genes associated with angiogenesis, erythropoiesis, and metabolism. While HIFs are essential for metabolic regulation in both adults and embryos, they also play key roles in pathologic processes such as tumorigenesis, metastasis, tumor-associated angiogenesis, and congenital erythrocytosis [1]. Germline and somatic mutations in genes involved in the hypoxia-sensing pathway, including Von Hippel-Lindau (VHL) and Egl-9 homolog 1 (EGLN1; also known as prolyl hydroxylase domain-containing protein 2 [PHD2]), have been described in neuroendocrine tumor syndromes associated with polycythemia [2–6]. Recently, somatic gain-of-function mutations in the oxygen-dependent domain (ODD) of HIF-2α were discovered in the context of multiple paraganglionomas, somatostatinomas, and polycythemia [7–9]. Dysregulation of the oxygen-sensing pathway occurs in many neuroendocrine tumors, but the exact mechanisms by which this dysregulation promotes oncogenesis remain largely unknown.

The liver is a primary site of hematopoiesis in embryos and extramedullary hematopoiesis in adults [10], and circulating hematopoietic stem cells may be capable of hepatocyte differentiation [11]. Polycythemia has been observed in patients with hepatocellular carcinoma (HCC) and is thought to result from the tumor’s secretion of erythropoietin [12, 13]. HCC is a relatively rare disease with an incidence between 3 and 18 per 100,000 in the United States [14, 15], but it may be as frequent as 24 per 100,000 in endemic countries [16]. Major risk factors for HCC include infection with oncogenic hepatitis B virus as well as chronic hepatitis due to hepatitis C virus infection, alcoholism, smoking, non-alcoholic fatty liver disease, and aflatoxin B1 intake [17–24]. In light of recent findings that *HIF2A* mutations can cause polycythemia in certain tumor syndromes, polycythemia in HCC might also involve genetic alternations in hypoxia-induced signaling pathways. In this study, we therefore sought to identify mutations in *HIF2A* in HCC patients presenting with polycythemia. We also examined in depth a previously undescribed germline c.G1645A (p.E549K) mutation in a critical oxygen-sensing region identified in one of the 302 HCC patients screened; this patient also exhibited polycythemia that resolved upon tumor resection.

## RESULTS

### HIF2A mutational analysis in HCC patients

Three hundred and two HCC patients underwent genomic DNA sequencing to identify mutations in *HIF2A*. Of the 302 patients, 104 exhibited polycythemia, while the remaining 198 patients did not. A unique heterozygous c.G1645A (p.E549K) mutation in the *HIF2A* gene was identified in the tumor DNA of only one patient (index patient) who exhibited concurrent polycythemia.

### Clinical history of index patient

**Figure 1:**
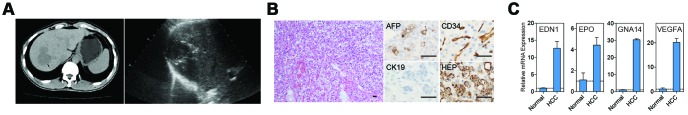
**Imaging, histopathology, and mutant HIF2A signaling in the index patient.** (**A**) Abdominal CT imaging and ultrasound revealed a hypodense and hypoechoic mass in the patient’s right liver. (**B**) Hematoxylin and eosin staining of the resected tumor revealed hepatocellular carcinoma, clear cell variant, with prominent angiogenesis. Tumor cells were positive for AFP and HEP and negative for CK19 by immunohistochemistry; endothelial cells stained positively for CD34. (**C**) Real-time PCR demonstrated overexpression of the HIF2A transcriptional targets *EDN1*, *EPO*, *GNA14*, and *VEGFA* within the tumor bed. Scale bars: 50 μm.

The index patient was a 43-year-old male who was referred to the Division of Hepatobiliary and Pancreatic Surgery clinic at the First Affiliated Hospital of Zhejiang University in August 2012 for a suspected hepatic hemangioma identified by abdominal computed tomography (CT). The patient presented with non-radiating abdominal pain on the right side below the xyphoid process that did not worsen with eating or changing position. He also complained of mild general weakness but denied any nausea or vomiting. Physical exam revealed no jaundice. Laboratory results are presented in Table 1. A viral hepatitis panel was positive for hepatitis B surface (HBs) antigen and antibodies to hepatitis B core antigen (HBc) and envelope antigen (HBe); there were no detectable antibodies to hepatitis C virus (HCV). Serum α-fetoprotein (AFP) was elevated at 89.5 ng/mL (reference range: < 20 ng/mL). A complete blood count (CBC) revealed a hemoglobin level of 188 g/L (reference range: 131-172 g/L), a red blood cell count of 5.8 × 10^12^/L (reference range: 4.09-5.74 × 10^12^/L), and a hematocrit of 54.2% (reference range: 38.0-50.8%). Abdominal ultrasonography revealed a hypo-echoic mass in the right liver and possible cholecystitis. A subsequent non-contrast abdominal CT revealed a 5.2 cm × 3.4 cm hypodense mass at the same location (Figure 1A). Hepatectomy was performed to remove the tumor.

**Table 1:** Laboratory values of the index patient and family members.

	**Index case**			**Sister (II-C)**	**Sister (II-E)**	**Niece (III-C)**	**Reference range**
	**Pre-operative**	**4 days post-operative**	**7 weeks post-operative**				
Serum α-fetoprotein (ng/ml)	89.5	Undetectable	Undetectable	2.9	ND	ND	< 20 ng/mL
Viral hepatitis panel				ND	ND	ND	
HBs Ag	Positive						
HBs Ab	Negative						
HBc Ab	Positive						
HBe Ag	Negative						
HBe Ab	Positive						
HCV Ab	Negative						
Complete blood count							
Erythrocytes × 10^12^/L	5.8	4.1	5.18	4.38	4.44	4.16	4.09 – 5.74
Hematocrit (%)	54.2	37.5	48.3	39.7	35.3	37.8	38.0 – 50.8
Hemoglobin (g/L)	188	135	165	143	115	133	131 – 172
Leukocytes × 10^9^/L	7.7	20.7	7.3	5.1	4.4	5.4	4 – 10
Platelets × 10^9^/L	227	186	178	217	379	314	83 – 303

The reference ranges presented are those used at the Massachusetts General Hospital.

The patient was stable post-operatively and his recovery was unremarkable. Histological examination of the tumor specimen revealed tumor cells with prominent clear cytoplasm arranged in a trabecular growth pattern (Figure 1B). Tumor cells were positive for AFP and Hep par 1 (HEP) stains but negative for cytokeratin 19 (CK19). The tumor also contained numerous capillary channels positive for CD34 staining. These observations supported a diagnosis of hepatocellular carcinoma, clear cell variant. On post-operative day four, CBC revealed a hemoglobin level of 135 g/L, a red blood cell count of 4.1 × 10^12^/L, and a hematocrit of 37.5%.

### Up-regulation of hypoxia-related gene expression

Expression of canonical hypoxia-induced genes in the tumor tissue of the index patient was examined using real-time PCR. Peritumoral liver tissue was used as a control. HIF-2α transcriptional targets *EDN1*, *EPO*, *GNA14*, and *VEGFA* were significantly upregulated in the tumor bed but not the surrounding liver tissue (Figure 1C), indicating hyperactivation of the hypoxia signaling pathway within the tumor.

### Germline mutational analysis in DNA of index patient and family

**Figure 2:**
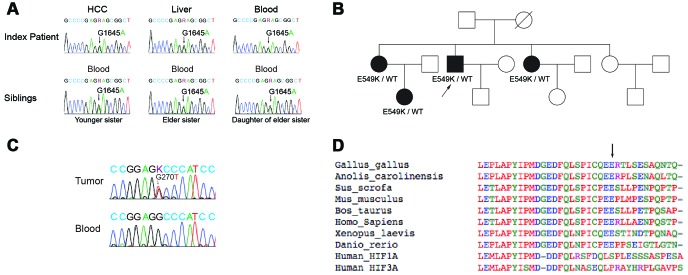
**Sanger sequencing, genetic pedigree, and localization of germline mutation in the index patient and family members.** (**A**) Direct Sanger sequencing results are shown for the *HIF2A* gene in the index patient and affected family members. The patient’s blood lymphocytes as well as the tumor and surrounding liver tissue carried a G1645A mutation in the gene coding for HIF2A. This mutation was also present in the blood lymphocytes of two of the patient’s sisters and one niece. (**B**) A family pedigree is shown. (**C**) Sanger sequencing of the *p53* gene in the tumor tissue and blood lymphocytes of the index patient is shown. The *p53* mutation was present only in the index patient’s tumor tissue. (**D**) Multiple peptide sequence alignment was performed; the E549 site was critically conserved across species (arrow). As such, a mutation at this site could potentially alter HIF-2α signaling.

To determine whether this was a somatic or germline mutation, additional *HIF2A* analyses were performed on DNA obtained from normal peritumoral tissue and blood lymphocytes of the index patient. Sanger sequencing confirmed the presence of the p.E549K mutation in both normal liver tissue and blood (Figure 2A), confirming a germline etiology.

Subsequently, additional studies were performed to identify this mutation in family members of the index patient. Blood lymphocytes from the proband’s family members revealed the p.E549K mutation was present in two sisters and one niece (Figure 2A). However, CBCs failed to demonstrate polycythemia in any family member, and none were diagnosed with HCC. These findings are illustrated in a genetic pedigree (Figure 2B).

### P53 mutational analysis in tumor of index patient

Because none of the index patient’s family members had been diagnosed with HCC, further studies were performed to identify any additional mutations that had accumulated in the tumor. Mutations in the p53 tumor suppressor gene are common in HCC [25–27]. As such, Sanger sequencing for *p53* was performed on DNA obtained from the tumor and blood lymphocytes of the index patient. A c.G270T (p.R90S) mutation was identified in the tumor DNA but not in the blood, confirming a somatic etiology (Figure 2C).

### Localization of HIF2A mutation

Having established a germline origin for the p.E549K mutation, additional studies were undertaken to elucidate the function of this genetic alteration. Multiple peptide sequence alignment demonstrated that E549 of HIF-2α is located proximal to the protein’s primary hydroxylation domain and is conserved across species (arrow, Figure 2D). An amino acid substitution at this site could therefore potentially affect the conformation of the HIF-2α hydroxylation center and alter its recognition by VHL.

### Stability of the mutant HIF-2α protein

**Figure 3:**
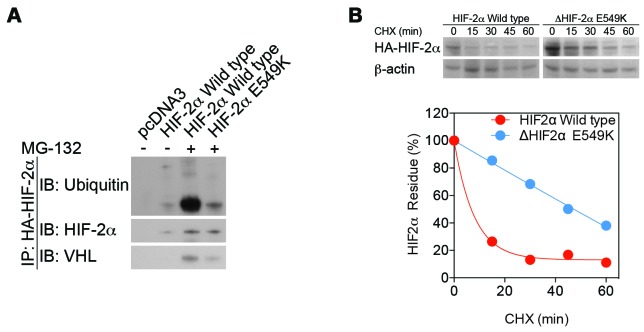
**Stability is increased, while enzymatic function is maintained, in mutant HIF-2α protein.** (**A**) An immunoprecipitation assay demonstrated lower affinity of mutant HIF-2α protein for binding to VHL and subsequent decreases in ubiquitination and proteasome degradation. (**B**) A cycloheximide assay confirmed that stabilization of mutant HIF-2α extended the half-life of the mutant protein.

To determine the effect of the c.G1645A mutation on HIF-2α protein stability, we performed an immunoprecipitation assay to analyze HIF-2α ubiquitination and von Hippel-Lindau protein (pVHL) recognition (Figure 3A). Under normoxic conditions, HIF-2α is hydroxylated and subsequently ubiquitinated by pVHL. The mutant E549K exhibited lower affinity for VHL, preventing its ubiquitination and subsequent degradation by the proteasome. A CHX assay confirmed that the stabilization of mutant HIF-2α extended the half-life of the mutant peptide (Figure 3B).

### Transcriptional activity of mutant HIF-2α

**Figure 4:**
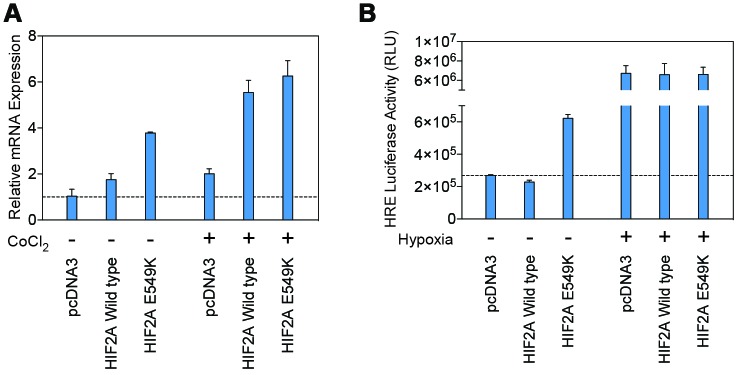
**Transcription of the mutant HIF-2α protein is maintained under normoxic and hypoxic conditions.** (**A**) Results from real-time PCR demonstrated similar transcription levels for the wildtype and mutant *HIF2A* genes under conditions of normoxia and simulated hypoxia. (**B**) A luciferase assay using Hep3B cells transfected with wildtype and p.E549K *HIF2A* confirmed these findings.

Transcription of the mutant HIF-2α peptide was determined using real-time PCR (Figure 4A) and a luciferase assay (Figure 4B). Transcription of mutant HIF-2α increased under normoxic conditions and was maintained during hypoxia, demonstrating that the p.E549K mutation results in a stable HIF-2α peptide without affecting its transcription.

## DISCUSSION

This report documents a novel germline gain-of-function *HIF2A* mutation in a family in which one individual developed HCC and polycythemia. Interestingly, the germline mutation itself was not associated with erythrocytosis; none of the affected family members who did not develop HCC had a history of polycythemia or associated symptoms. Furthermore, although all cells carried the mutation, only tumor cells from the index patient exhibited upregulation of the transcriptional targets of HIF-2α. Removal of the tumor resolved his polycythemia.

Several *HIF2A* mutations are known to cause polycythemia. All of them result in amino acid substitutions or in-frame deletions in the primary hydroxylation domain, thereby subverting normal oxygen sensing and driving tumor formation [7–9]. The first such mutation discovered was p.G537W, which was originally reported as a germline mutation found across three generations in a single family that causes Familial Erythrocytosis Type 4 per Online Mendelian Inheritance in Man (OMIM) [28]. Since then, numerous somatic gain-of-function mutations have been identified, including p.P534L, p.M535V, p.M535I, p.G537W, p.G537R, and p.D539E, all of which are located in the ODD of *HIF2A* [29]. Like these mutations, the p.E549K mutation lies in the evolutionarily-conserved primary hydroxylation site of HIF-2α. This precludes oxygen-dependent HIF-2α hydroxylation by PHD enzymes, ubiquitination by the pVHL-E3 ligase complex, and subsequent proteasomal degradation, resulting in constitutive hypoxia signaling in the presence of normal oxygen tension.

More recently, *HIF2A* mutations have been implicated in unique clinical syndromes in patients with neuroendocrine tumors associated with polycythemia. These include the somatic mutations p.A530V [30] and p.A530E [31], somatic mosaic mutations p.L529P and p.L542P [32], and the germline mutation p.F374Y [33]. While HIF2A overexpression promotes oncogenesis in renal cell carcinoma [34], the p.E549K mutation described in the index patient here is the first *HIF2A* mutation to be identified in HCC. Previous studies have shown that prolonged HIF activation may accelerate the development of fibrosis, promote viral replication in chronic liver disease, and promote tumor cell growth and metastasis [35–38]. Hepatocellular carcinoma is typical of metabolically heterogeneous cancers in that tumor cells often generate energy through aerobic glycolysis rather than relying on oxidative phosphorylation in the mitochondria, even when oxygen supplies are adequate [23, 24]. This phenomenon, known as the Warburg effect, promotes tumor cell growth not only by providing an energy source for rapid cell proliferation, but also by minimizing the production of reactive oxygen species [39, 40]. The p.E549K mutation observed here stabilized the mutant peptide, which may transcriptionally up-regulate many genes encoding glycolytic enzymes [39]. Additionally, the HIF2α transcriptional targets EDN1, EPO, GNA14, and VEGFA were significantly upregulated in the tumor and might also promote tumor progression. Interestingly, despite its germline inheritance, the p.E549K mutation upregulated HIF-2α targets in the tumor but not the surrounding tissue, indicating that it selectively activated hypoxia signaling within the tumor. Moreover, the lack of obvious necrosis in the tumor suggests aberrant hypoxia signaling. Why *HIF2A* expression was not upregulated in the patient’s normal cells and why the other affected family members did not exhibit polycythemia remain unclear. These findings suggest that this mutation may show incomplete penetrance in causing the polycythemia phenotype. Well-known examples of gain-of-function mutations with incomplete penetrance and tumor predisposition include *RET* mutations in multiple endocrine neoplasia (MEN) syndromes and *GNAS1* mutations in McCune-Albright syndrome. Like these syndromes, tumor development arising from a germline p.E549K mutation likely requires additional mutagenic changes, such as the *p53* mutation reported in the index patient.

The mechanisms underlying potential carcinogenic interactions between the *p53* tumor suppressor pathway and HIF expression are not fully understood. HIF expression is associated with increased genomic instability [41] and can promote malignant transformation through antagonism of p53-mediated tumor suppression [42, 43]. In clear-cell renal cell carcinoma, for instance, HIFs act upon the p53 pathway to mediate the DNA damage response [44], and overexpression of *HIF2A* suppresses Hdm2-mediated p53 activity, conferring chemotherapeutic resistance [43]. Likewise, inhibition of HIF-2α activity abrogates p53 activity, significantly sensitizing clear-cell renal carcinoma cells to radiation-induced apoptosis [45]. Therefore, further studies are needed to elucidate any potential synergy or causality between *p53* mutations and gain-of-function mutations in hypoxia induced signaling pathways. In the case of the index patient, it is unclear whether selective activation of *HIF2A* signaling was attributable to or further propagated by the *p53* mutation.

Given that the index patient’s polycythemia resolved upon total tumor resection, it is probable that the erythrocytosis in this case was due to upregulation of *HIF2A* targets by the novel mutant protein. However, this mutation was found in only one of 104 total HCC patients with concurrent polycythemia, suggesting that genetic alterations in *HIF2A* are involved in only a subset of HCC patients with erythrocytosis. There are numerous other possible etiologies for polycythemia, including mutations in PHD enzymes, VHL proteins, alterations in angiotensin II signaling, upregulation of insulin-like growth factor-1 signaling, and constitutive activation of the JAK/STAT pathway [46–50]. Additional investigations are needed to determine the molecular mechanisms and therapeutic significance of polycythemia in HCC patients.

## MATERIALS AND METHODS

### Patients

302 patients with HCC including 104 with polycythemia were referred to the Division of Hepatobiliary and Pancreatic Surgery, Department of Surgery at the First Affiliated Hospital of Zhejiang University and fulfilled the diagnostic criteria for hepatocellular carcinoma described by the American Association for the Study of Liver Diseases (AASLD) [51]. Patients with hematological system disorders were excluded from the study. Polycythemia was defined as an elevated hematocrit or red blood cell count that did not meet the 2007 World Health Organization (WHO) criteria for diagnosis of polycythemia vera [52].

### Study oversight

This study was approved by the institutional review board of the First Affiliated Hospital, College of Medicine, Zhejiang University (Reference number: 2014-272). Pedigree investigation participants provided written informed consent for participation in this study. Written informed consent was not obtained from other screened patients because this study used only preexisting data and pathological tissues; in these cases, we informed the participants of their right to refuse enrolment via telephone interview. These informed consent and enrolment procedures are in accordance with the detailed regulations described in the guidelines.

### Laboratory studies

### Immunohistochemistry

Formalin-fixed, paraffin-embedded tissue sections were probed with primary antibodies against AFP (mouse monoclonal; Zhongshan Golden Bridge Biotechnology Company Limited, Beijing, China), CD34 (mouse monoclonal; Long Island Biotechnology Company Limited, Shanghai, China), CK19 (mouse monoclonal; Zhongshan), or HEP (mouse monoclonal; Zhongshan). Slides were then stained with 3,3’-diaminobenzidine (DAB) and visualized using a Leica DM LB light (Solms, Germany) microscope at magnifications of 100× or 400× as indicated.

### Mutation analysis

Genomic DNA was extracted using the DNeasy Blood and Tissue Kit (Qiagen, Venlo, Netherlands). *HIF2A* exons were amplified by polymerase chain reaction (PCR) using previously described primer sets [7]. Likewise, *P53* exons were amplified by PCR using previously described primer sets [53]. Amplicon sequences were then determined by Sanger sequencing.

### Plasmids and mutagenesis

A pcDNA3 plasmid (Addgene, Cambridge, MA; plasmid 18950 [7]) containing the human *HIF2A* coding sequence was used for wild-type HIF2A expression analysis. E549K-mutant *HIF2A* was cloned and inserted into an expression plasmid using a Quikchange Lightning Site-directed Mutagenesis Kit (Agilent Technologies, Santa Clara, CA). Sanger sequencing was used to confirm successful insertion.

### Real-time PCR

Total RNA was extracted from microdissected tumor samples using an RNeasy Plus Mini Kit (Qiagen). Messenger RNA (mRNA) expression of HIF target genes was examined by real-time polymerase chain reaction (PCR) using an Eco Real-Time PCR System (Illumina, San Diego, CA). Gene-specific primer sets included endothelin-1 (EDN1; Qiagen QT00088235), erythropoietin (EPO; Origene, Rockville, MD; HP200740), guanine nucleotide binding protein alpha 14 (GNA14; Qiagen QT00099379), vascular endothelial growth factor A (VEGFA; Qiagen QT01682072), and beta-actin (ACTB; Qiagen QT00095431).

### Immunoprecipitation

*HIF2A*-transfected HEK293 cells were treated with MG-132 (10 μM) for 6 hr. Total protein was extracted using immunoprecipitation (IP) lysis buffer supplemented with a protease inhibitor cocktail (Thermo, Waltham, MA). According to the manufacturer’s instructions, 500 μg total protein extract was incubated with 3 μg monoclonal anti-hemagglutinin (HA) antibody (Covance, Princeton, NJ) and a Dynabeads Protein G Immunoprecipitation Kit (Life Technologies, Carlsbad, CA) overnight at 4°C.

The eluent was then subjected to Western blot and probed with antibodies for ubiquitin (Abcam, Cambridge, UK), HIF-2α (Novus Biologicals, Littleton, CO), or VHL protein (Cell Signaling Technology, Danvers, MA).

### Cycloheximide assay

*HIF2A*-transfected HEK293 cells were treated with cycloheximide (CHX) (50 μM, Sigma-Aldrich, St. Louis, MO) for 0, 15, 30, 45, or 60 min as indicated. Total protein was extracted using RIPA lysis buffer supplemented with a protease inhibitor cocktail (Thermo). HIF-2α protein levels were then determined by Western blot using an anti-HA antibody (Covance).

### Luciferase assay

Hep3B cells stably expressing HRE-luciferase (Promega, Madison, WI) were transfected with *HIF2A* recombinants. Cells were incubated in 21% or 1% O_2_ for 12 hr and luciferase activity was determined using the ONE-Glo Luciferase assay system (Promega).
